# Microstrain screening towards defect-less layered transition metal oxide cathodes

**DOI:** 10.1063/4.0001041

**Published:** 2025-10-27

**Authors:** Tianyi Li

**Affiliations:** Argonne National Laboratory, Lemont, IL, USA

## Abstract

Microstrain and the associated propagation of structural defects from the surface to the bulk are significant challenges in developing high energy, long life energy storage systems. However, the origins and impact of microstrain during the material synthesis remain poorly understood. In this study, we conducted real time, in situ microstrain screening during the synthesis of layered oxide cathode materials using multiscale synchrotron X-ray diffraction and microscopy techniques. Our findings reveal that the spatial distribution of transition metals within individual precursor particles critically influences nanoscale phase transformations, local charge heterogeneity, and the accumulation of microstrain. This unexpected dominance of transition metals results in a counterintuitive outward propagation of defect nucleation and growth. These insights provide a pathway for a more rational synthesis approach to minimize microstrain and crystallographic defects, enhancing the structural stability of battery materials. This work marks an essential step toward synthesis-by-design of defect-free materials for energy storage systems.

